# Analysis of the artificial vaginal microecology in patients after laparoscopic peritoneal vaginoplasty

**DOI:** 10.1038/s41598-019-44511-w

**Published:** 2019-06-11

**Authors:** Chenglu Qin, Guangnan Luo, Xin Luo, Brian N. Lifschutz, Ziwen Zhu, Yujiang Fang

**Affiliations:** 1 Departments of Gynecology and Obstetrics, The First Affiliated Hospital of Jinan University, Guangzhou, China; 2 Departments of Gynecology and Obstetrics, Luohu Hospital, Shenzhen, China; 3 Department of Microbiology, Immunology & Pathology, Des Moines University, Des Moines, Iowa USA; 4 Department of Surgery, University of Missouri School of Medicine, Columbia, MO USA

**Keywords:** Infertility, Sexual dysfunction

## Abstract

To investigate the artificial vaginal microecological features in patients who underwent laparoscopic peritoneal vaginoplasty. 54 cases of patients with artificial vagina after laparoscopic peritoneal vaginoplasty were included in this study. Microecosystem evaluation was performed. Artificial vaginal functional tests and biopsy from vaginal walls were performed. After laparoscopic peritoneal vaginoplasty, the artificial vaginal flora intensity was level II∼III (88.9%); the vaginal flora diversity was level II∼III (72.2%); the predominant vaginal bacteria were gram-positive macrobacillus (27.8%); approximately 57.4% of the patients had vaginal pH ≤ 4.5; there was no pathogenic bateria or other pathogens; dysbiosis accounted for 53.7% of the patients (64.5% of the patients who had the vaginoplasty operation less than 2 years ago exhibited dysbiosis; 39.1% of the patients who had the operation at least 2 years ago exhibited dysbiosis). Vaginal dysbiosis is common after laparoscopic peritoneal vaginoplasty. However, as time goes by, the artificial vaginal microecological condition gradually becomes normal. Evaluation of vaginal microenvironment after laparoscopic peritoneal vaginoplasty might play an important role in reproductive tract infection prevention and neovagina health care.

## Introduction

Many species of microbes reside in the normal vagina in physiological conditions, forming a normal vaginal microecology. However, the vaginal microecology is dynamic and is easily changed easily in adaptation to some endogenous and exogenous factors^1–3^. Compared to natural normal vagina, the artificial vagina reconstitued through laparoscopic peritoneal vaginoplasty has similar anatomical and physiological funcctions such as adequate depth and width, proper tissue elasticity, satisfactory cosmetic outlooking of the vulva. However, nothing is known about the flora of the artifical vaginal microecology after vaginoplasty. This study is designed to investigate the vaginal microecological condition after laparoscopic peritoneal vaginoplasty.

## Methods

### Objects

This study was conducted at the Luohu Hospital, Shenzhen, China. Fifty-four patients were included in this study. Laparoscopic peritoneal vaginoplasty was performed in our hospital to treat the congenital absence of vagina between July 2015 and January 2018. The postoperative follow up periods ranged from 4 months to 141 months (average 29 months). The ages of patients ranged from 18 to 39 years (average 29 years old). The patients were classified into two groups according to the postoperative period: postoperative period ≥2 years (average period 56.7 months, 23 patients), postoperative period <2 years (average period 10.1 months, 31 patients). This study was carried out according to the Helsinki Declaration and written informed consent was obtained from all subjects. This study was approved by the ethics committee of the Luohu Hospital (ID: 2018LHYYFCK-001-01).

#### Microecosystem evaluation

Samples of vaginal discharge were obtained from the upper one third of vaginal wall at the time for their followup (average period 56.7 months for over 2 year group vs. 10.1 months for below 2 year group). Some samples were placed on a slide; light microscopy was then used to directly evaluate cleanliness. Others of the samples were loaded onto another slide; gram staining was performed and the amount of lactobacillus, gardnerella, candida, trichomonas, streptobacillus, leukocytes, gonococci, etc. was evaluated under microscope; precision pH strips ranging from 3.8 to 5.4 and digital pH meter were used to measure the pH of vagina.

#### Artificial vaginal functional tests and biopsy from vaginal walls

The length, width, elasticity, moisture of the artificial vagina and the quality of sex life were all evaluated, and then a biopsy was performed. The size of the tissue removed from vaginal wall was 0.5 cm*0.5 cm.

#### Diagnostic criteria of vaginal microecology

Evaluation of artificial viginal miecroecology is based on previous study by using vaginal flora intensity, vaginal flora diversity, dominant bacterium, etc.^4^. Vaginal flora intensity: the average number of bacteria in the microscopic field under oil immersion lens and divided into four levels: level I, 1–9/field; level II, 10–99/field; level III, >100/field; and level IV, clustered bacteria or bacteria densely covering mucosal epithelial cells. Vaginal flora diversity: the number of different bacterial flora that could be identified in the microscopic field under oil immersion lens and divided into four levels: level I, 1–3 types/field; level II, 4–6 types/field; level III, 7–10 types/ field; and level IV, >11 types/field. Dominant bacterium: the most frequently observed microorganism in the microscopic field. Causative microorganism: the more frequent presence of either fungal hyphae or trichomoniasis. Vaginal pH: normal, pH ≤ 4.5; abnormal, pH > 4.5.

#### Diagnostic classification

(a) Normal Microecology: Vaginal flora intensity, II∼III level; vaginal flora diversity, II∼III level; gram-positive macrobacillus as the predominant bacterium; pH ≤ 4.5; H2O2 positive. (b) Dysbiosis: abnormality in any of the vaginal flora intensity, vaginal flora diversity, predominant bacterium, pH, or H2O2.

### Statistical analysis

SPSS software (version 11.0) was used for the statistical analysis. The age was expressed as mean ± SD. The χ^2^ test was used for comparing proportions and indicated statistical significance when p-value was smaller than 0.05.

## Results

### Vaginal physical condition and microecology

The vaginal appearances of all 54 patients were normal. The biopsy of artificial vagina was performed from the middle part of vaginal walls. The results showed normal stratified squamous epithelia as normal vagina. The 54 patients had vaginal length ranging from 8 to 12 cm (average length of 9.6 cm) and width of 2–3 fingers. All patients had good vaginal moisture and elasticity and leucorrhea-like vaginal discharges. 45 patients were sexually active, and 41 patients were satisfied with their sex lives. The proportion of satisfaction was 91%.

### The microecological evaluation showed microecological abnormalities occurring in a great proportion of patients

The predominant gram-positive macrobacillus were detected in 15 patients of 54 patients (27.8%); the pH values of 31 patients of 54 patients were ≤4.5 (57.4%). No pathogenic microorganism was detected in all 54 patients. Flora abnormalities mostly included flora suppression (low intensity or diversity); gram-positive microbacillus as the predominant bacterium was found in 12.3% of patients; gram-positive coccus as the predominant bacterium accounted for 31.5%; gram-negative bacillus as the predominant bacterium accounted for 9.6%. In 54 patients, none of the flora intensity was level I and in 15 patients (38.4%) the vaginal flora diversity was categorized as level I. In those 15 patients, 9 had undergone the operation equal to or greater than 2 years before the postoperative follow-up with microecological detection, and another 6 patients had undergone the operation less than 2 years before the follow-up (Table 1).

**Table 1:** Artifical vaginal microecological status after laparoscopic peritoneal vaginoplasty in 54 patients.

	Cases (n)	Proportion (%)
**pH**		
≤4.5	31	57.4
>4.6	23	42.6
**Vaginal flora intensity**		
I level	6	11.1
II level	18	33.3
III level	30	55.6
IV level	1	1.86
**Vaginal flora diversity**		
I level	15	27.8
II level	39	72.2
III level	0	0
IV level	0	0
**Predominant bacterium**		
Gram + macrobacillus	15	27.8
Gram + microbacillus	8	14.8
Gram- bacillus	21	38.9
Gram + coccus	10	18.5
**Pathogenic microorganism**		
Fungi	0	0
Trichomoniasis	0	0
BV	0	0
**Cleanliness**		
I level	0	0
II level	29	53.7
III level	22	40.7
IV level	3	5.6%
**Results of microecological detection**		
Dysbiosis	29	53.7%
Normal Microecology	25	46.3%

### The association between the artificial vaginal microecology and the postoperative time

#### Comparison of the vaginal pH, predominant bacterium and cleanliness between the two patient groups with different postoperative times

Comparing the vaginal pH between two patient groups with the different postoperative times, the proportion of patients (postoperative time ≥2 years) who had a normal vaginal pH was obviously larger than that of the patients (postoperative time <2 years) who had a normal vaginal pH. According to the χ^2^ test (χ^2^ = 9.01, P < 0.05), there was a significant difference. The proportion of the patients (postoperative time ≥2 years) who had gram-positive bacillus as the predominant bacterium was larger than that of the patients (postoperative time <2 years) who had gram-positive bacillus as the predominant bacterium. However, the χ^2^ test (χ^2^ = 0.98, P > 0.05) indicated there was no significant difference. The patients (postoperative time ≥ 2 years) had a larger proportion of high level vaginal cleanliness (Table 2).

**Table 2:** The association between the pH of vagina and the postoperative period of the patients.

	Cases (%) (postoperative time ≥2 years)	Cases (%) (postoperative time <2 years)
**Vaginal pH**		
pH > 4.5	4 (17.4%)	18 (58.1%)
pH ≤ 4.5	19 (83.6%)	13 (42.9%)
**Predominant bacterium**		
Gram + macrobacillus	8 (38.1%)	7 (22.6%)
Gram + microbacillus	3 (14.3%)	5 (16.1%)
Gram- bacillus	10 (43.5%)	11 (35.5%)
Gram + coccus	2 (8.7%)	8 (25.8%)
**Vaginal cleanliness**		
I level	0	0
II level	19 (82.6%)	10 (32.3%)
III level	3 (13.0%)	19 (61.3%)
IV level	1 (4.3%))	2 (6.5%)

#### Comparison of the vaginal flora intensity between the two patient groups with different postoperative times

The proportion of patients (postoperative time ≥2 years) who had normal vaginal flora intensity (level II-III) was slightly larger than that of patients (postoperative time <2 years) who had normal vaginal flora intensity; the proportion of patients (postoperative time ≥2 years) who had normal vaginal flora diversity (level II-III) was smaller than that of patients (postoperative time <2 years) who had normal vaginal flora diversity, however, there was no significant difference (Table 3, X^2^ = 2.57, P > 0.05).

**Table 3:** Comparison between the vaginal flora intensity and the vaginal flora diversity of the patients.

Case (%)	I level	II level	III level	IV level
Vaginal flora intensity (postoperative time ≥2 years)	2 (8.9%)	6 (26.1%)	15 (65.2%)	0
Vaginal flora intensity (postoperative time <2 years)	4 (12.9%)	11 (35.5%)	16 (51.6%)	0
Vaginal flora diversity (postoperative time ≥2 years)	9 (39.1%)	14 (60.9)	0	0
Vaginal flora diversity (postoperative time <2 years)	6 (19.4%)	25 (80.6%)	0	0

#### Comparison of the vaginal dysbiosis between the two patient groups with different postoperative times

In 23 patients (postoperative time ≥2 years) 60.9% (14) exhibited normal microecology, and 39.1% of the patients (9) exhibited dysbiosis. In 31 patients (postoperative time <2 years), 35.5% of the patients (11) had normal microecology, and 64.5% of patients (20) exhibited dysbiosis. As postoperative time went by, the proportion of patients having normal microecology increased, however there was no evidence showing significant difference (Table 4).

**Table 4:** The proportion of the patients having dysbiosis.

Case (%)	Normal microecology	Dysbiosis
postoperative time ≥2 years	14 (60.9%)	9 (39.1%)
postoperative time <2 years	11 (35.5%)	20 (64.5%)
X^2^	3.42	
P-value	>0.05	

## Discussion

The study of vaginal microecology has become hot in recent years. Increasing evidence suggests that some common clinical gynecological infectious diseases as well as some tumors are closely associated with vaginal dysbiosis^5–8^. Thus, the study of vaginal microecology is of great clinical significance. The artificial vagina through laparoscopic peritoneal vaginoplasty has similar anatomical and physiological features compared to a normal vagina. however, there was no microbes in the artificial vagina theoretically, so the study of the vaginal microecology might play a positive role in improving the health management of the artificial vagina.

The vaginal microecosystem consists of vaginal anatomical structure, flora, a local immune system, and an endocrine system^9–11^. It is a special, dynamic system whose normal vaginal flora is the core content of the study of vaginal microecology. According to the research results of normal vaginal microecology by Zhuohui Liu, normal vaginal flora intensity is usually level II- III, accounting for 97.7%; vaginal flora diversity is level II- III, accounting for 94.6%; the predominant bacterium as gram-positive macro-bacillus (lactobacillus) accounts for 99.9%; the majority has a vaginal pH < 4. 5, accounting for 72.1%; 2/3 of the females without vaginal infection exhibit normal vaginal microecology, but a small proportion of women exhibit abnormalities of vaginal microecology. Based on our study, the artificial vagina generated by laparoscopic peritoneal vaginoplasty had good physical functions, and the patients after their operation were satisfied with their sex life; however, dysbioses were found in a large proportion of the artificial vaginas. The patients with normal microecology (25) accounted for 46.3% of patients (54); the flora intensity was mostly normal (level II- III) in 88.9% of the cases and the flora diversity was mostly normal (level II- III) in 72.2% of the cases. Despite the large proportions of normal flora intensity and of normal flora diversity, the predominant bacterium as gram-positive macrobacillus (lactobacillus) only accounted for 27.89% and 57.4% of patients had a vaginal pH ≤ 4.5. The proportions were smaller than the research results of the vaginal microecology of healthy people^10^. The proportion of normal vaginal microecologies among the artificial vaginas was different from the report of the proportion of the vaginal microecology of normal people^10^, but similar to the report by Rui He^11^. In this study, the proportions of the normal vaginal flora intensity and that of the normal vaginal flora diversity were relatively large, though the proportion of normal vaginal microecology was small due to the small proportion of lactobacilli as the predominant bacteria. Thus, although an artificial vagina functions similarly to a normal one, an artificial vagina is still different from normal in terms of microecology, especially considering the small proportion of lactobacilli as the predominant bacteria. Hence, it is one of important contents in the future of vaginal health management after laparoscopic peritoneal vaginoplasty to establish the normal vaginal microecology of the artificial vagina.

The vaginal microecological system changes with the changes of physiological status and local factors, and furthermore, the predominant bacterium varies from place to place and among races as well. Patients with the congenital absence of a vagina have normal ovarian function and estrogen fluctuations. In the early stages after the operation, the artificial vagina belongs to a “blank” niche in terms of vaginal microecoloy. However, with the stimulation of estrogen, mold, expansion after the operation, cleaning and sex life, the vagina changes from a nonbacterial status to a bacterial status, from dysbiosis to normality, with a special revolution of microecology. According to our study, the vaginal microecology was related to the postoperative period. The results of the biopsy indicated that the artificial vagina was completely covered by stratified squamous epithelium and had the same tissue features of a normal vagina. As time went by, the artificial vagina matured and the vaginal microecology tended to be normal. Using 2 years after the operation as a cutoff point, a pH ≤ 4.5 was exhibited in 19 (82.6%) of 23 patients (postoperative period ≥ 2 years), as well as in 18 (58.1%) of 31 patients (postoperative period <2 years). 11(35.5%) of 31 patients (postoperative period <2 years) had normal microecology and normal microecology was exhibited in 14 (60.9%) of 23 patients (postoperative period ≥ 2 years). The comparison of the proportions showed significant difference. Normal vaginal cleanliness accounted for 82.6% of patients (postoperative period ≥ 2 years) and 32.3% of patients (postoperative period <2 years). Furthermore, normal predominant bacterium was found in 8 (34.8%) of 23 patients (postoperative period ≥ 2 years) and in 7 (22.6%) of 31 patients (postoperative period <2 years).

From our study, it is indicated that as postoperative time went by, the proportion of patients with gram-positive macrobacillus as the predominant bacterium increased. The reason may be that in the early stages after the operation, the thin vaginal epithelium reduces the production of glycogen, leading to the reduction of lactic acid and the increase of pH, which inhibits the growth of lactobacillus. As postoperative time went by, vaginal epithelial cells went back to normal, increased the production of glycogen and lactic acid, which promoted the growth of lactobacilli. Consequently, the proportions of normal pH and lactobacilli as the predominant bacteria increased after a 2-year postoperative period. According to our study, the formation of the normal microecology in an artificial vagina promotes squamous epithelialization and boosts the growth of lactobacilli.

In this study, factors listed in Table 1 at different time points after surgery from the same patient such as at 2, 4, 6, 12, 18 and 24 months after surgery could not be provided because almost all patients were from nationwide and they usually chose to keep their privacy as much as they could by avoiding postsurgical follow up. In recent two years, limited data for a few patients has been recorded, unfortunately, it is not enough for statistics.

It might be necessary to point out that it is unclear whether age affects postoperative changes in postoperative vaginal microecology. In fact, all patients in this study were operated after puberty with a normal ovary function and normal levels of female hormones. Peritoneal vaginoplasty was performed by using sterile peritoneum to cover the artificial vagina. Accordingly, it is reasonable that the artificial vagina after peritoneal vaginoplasty should be aseptic within the first couple of days after surgery. Thus, age might not be an important factor regarding the maintenance of artificial vaginal microecology.

In short, the artificial vagina created by laparoscopic peritoneal vaginoplasty has its own special flora of vaginal microecology. As postoperative time goes by, the microecological condition gradually becomes normal and the proportion of gram-positive macrobacillus as the predominant bacterium increased. To understand the flora of the vaginal microecological condition after laparoscopic peritoneal vaginoplasty and explore appropriate adjunctive therapies, it will be beneficial to establish a normal vaginal microecology in an artificial vagina.

## Conclusions

In patients with congenital vaginal atresia who have undergone laparoscopic peritoneal vaginoplasty, the vaginal microecology generated exhibits dysbiosis in varying degrees. Most forms of the dysbiosis include the increase of vaginal pH, the lack of vaginal flora diversity, and the small proportion of normal predominant bacterium. The flora of vaginal microecology after laparoscopic peritoneal vaginoplasty was associated with the postoperative period, and the proportion of normal microecology in the patients with an artificial vagina increased as postoperative time went by.

## Data Availability

The datasets used and/or analyzed during the current study are available from the corresponding author on reasonable request.
